# Hypertabastic survival model

**DOI:** 10.1186/1742-4682-4-40

**Published:** 2007-10-26

**Authors:** Mohammad A Tabatabai, Zoran Bursac, David K Williams, Karan P Singh

**Affiliations:** 1 Department of Mathematical Sciences, Cameron University, Lawton, OK, USA; 2 Department of Biostatistics, University of Arkansas for Medical Sciences, Little Rock, AR, USA; 3 Department of Biostatistics, University of North Texas Health Science Center, Ft.Worth, TX, USA

## Abstract

A new two-parameter probability distribution called hypertabastic is introduced to model the survival or time-to-event data. A simulation study was carried out to evaluate the performance of the hypertabastic distribution in comparison with popular distributions. We then demonstrate the application of the hypertabastic survival model by applying it to data from two motivating studies. The first one demonstrates the proportional hazards version of the model by applying it to a data set from multiple myeloma study. The second one demonstrates an accelerated failure time version of the model by applying it to data from a randomized study of glioma patients who underwent radiotherapy treatment with and without radiosensitizer misonidazole. Based on the results from the simulation study and two applications, the proposed model shows to be a flexible and promising alternative to practitioners in this field.

## 1. Introduction

Time to event models, commonly known as survival or reliability models, have been studied and applied in a variety of scientific disciplines such as medicine, engineering and business. The Hosmer and Lemeshow [1], Lee and Wang [2], Kleinbaum and Klein [3], and Collet [4] books give a detailed overview of survival data modeling techniques. Non-parametric and semi-parametric survival models such as the Cox regression analysis have been the most widely used models in the analysis of time to event survival data [5]. On the other hand, if the assumption for parametric probability distribution is met for the data set under consideration, it will result in more efficient and easier to interpret estimates than non-parametric or semi parametric models. A comprehensive review was given by Efron [6] and Lee and Go [7].

Parametric hazard functions can enable clinicians and researchers to model various disease scenarios, assess disease prognosis and progression, give valuable insights on the pattern of failure, and understand the pathogenesis of a chronic disease and how they are affected by different treatment effects [8-10]. Estimation of hazard function is also useful in the analysis of change-point hazard rate models. It helps policy makers with cost effective health care policy decisions [11]. Lundin et al. [12] estimated the survival probabilities in breast cancer patients and concluded that parametric survival estimates may be more precise than Kaplan-Meier estimates when there are few patients in a particular stratum. Royston and Parmar [13] modeled the baseline distribution function by restricted cubic regression spline. Kay et al. [14] discussed the use of hazard functions in breast cancer studies. They believe that the hazard function is an important tool in investigating disease curability and can help the clinician to express his ideas regarding disease progression and the biology of treatment effect. Foulkes et al. [15] used parametric modeling to assess the prognostic factors in the recurrence of ischemic strokes. Sama et al. [16] used five parametric models to analyze the survival time data of infections and they found that the best fit could be obtained using parametric models. They also indicated that parametric models can be used to model the duration of malaria infections. Kannan et al. [17] used log-logistic probability distribution to model altitude decompression sickness (DCS) risk and symptom onset time. They concluded that the log-logistic model could provide good estimates of the probability of DCS over time. Nardi and Schemper [18,19] emphasized the role of residuals for the selection of survival models. They argued that when empirical data is sufficient, parametric models provide some insight into the shape of the baseline hazard function.

In the Cox model, the baseline hazard function is regarded as a nuisance parameter, while in parametric models, the hazard function reflects the time course of the process under study. In this paper, we introduce a new two-parameter continuous probability distribution called hypertabastic probability distribution. The hypertabastic hazard function can assume a different variety of shapes. It can be used to analyze biomedical data such as cancer recurrence time. Based on the hypertabastic distribution, we introduce the hypertabastic survival model which includes the hypertabastic proportional hazards model with parametric baseline hazard function, the hypertabastic accelerated failure model and the hypertabastic proportional odds model. The hypertabastic distribution can be used to analyze the accelerated hazards regression model of Chen [20]. It can also be used to monitor the disease progression and regression and provide the clinicians with the time interval(s) on which the disease progresses or regresses and the time interval(s) on which the disease progression or regression speeds up or slows down. This vital information will make it easier for the physicians to take appropriate action regarding their patients.

## 2. Hypertabastic distribution

In this section we introduce a new probability distribution which can be used in many scientific disciplines. Such disciplines may include, but are not limited to, biomedical, engineering, and business fields.

Definition 2.1 (Hypertabastic Distribution) We say a continuous random variable *T *has a hypertabastic distribution if its cumulative distribution function is

F(t)={1−Sech[α(1−tβCoth(tβ))/β]for t>00for t<0
 MathType@MTEF@5@5@+=feaafiart1ev1aaatCvAUfKttLearuWrP9MDH5MBPbIqV92AaeXatLxBI9gBaebbnrfifHhDYfgasaacH8akY=wiFfYdH8Gipec8Eeeu0xXdbba9frFj0=OqFfea0dXdd9vqai=hGuQ8kuc9pgc9s8qqaq=dirpe0xb9q8qiLsFr0=vr0=vr0dc8meaabaqaciaacaGaaeqabaqabeGadaaakeaacqWGgbGrcqGGOaakcqWG0baDcqGGPaqkcqGH9aqpdaGabeqaauaabaqaciaaaeaacqaIXaqmcqGHsislcqWGtbWucqWGLbqzcqWGJbWycqWGObaAdaWadaqaaGGaciab=f7aHjabcIcaOiabigdaXiabgkHiTiabdsha0naaCaaaleqabaGae8NSdigaaOGaem4qamKaem4Ba8MaemiDaqNaemiAaG2aaeWaaeaacqWG0baDdaahaaWcbeqaaiab=j7aIbaaaOGaayjkaiaawMcaaiabcMcaPiabc+caViab=j7aIbGaay5waiaaw2faaaqaaiabbAgaMjabb+gaVjabbkhaYjabbccaGiabdsha0jabg6da+iabicdaWaqaaiabicdaWaqaaiabbAgaMjabb+gaVjabbkhaYjabbccaGiabdsha0jabgYda8iabicdaWaaaaiaawUhaaaaa@62A1@

The parameters α and β are both positive and *Sech *[•] and *Coth *[•] are hyperbolic secant and hyperbolic cotangent respectively. We often read as "*T *is hypertabastically distributed with parameters *α *and *β "*and write it as *H *(*α*, *β*). The probability density function of *T *is given by

f(t)={Sech[W(t)](αt2β−1Csch(tβ)2−αtβ−1Coth(tβ))Tanh[W(t)]for t>00for t<0
 MathType@MTEF@5@5@+=feaafiart1ev1aaatCvAUfKttLearuWrP9MDH5MBPbIqV92AaeXatLxBI9gBaebbnrfifHhDYfgasaacH8akY=wiFfYdH8Gipec8Eeeu0xXdbba9frFj0=OqFfea0dXdd9vqai=hGuQ8kuc9pgc9s8qqaq=dirpe0xb9q8qiLsFr0=vr0=vr0dc8meaabaqaciaacaGaaeqabaqabeGadaaakeaacqWGMbGzcqGGOaakcqWG0baDcqGGPaqkcqGH9aqpdaGabeqaauaabaqaciaaaeaacqWGtbWucqWGLbqzcqWGJbWycqWGObaAdaWadaqaaiabdEfaxnaabmaabaGaemiDaqhacaGLOaGaayzkaaaacaGLBbGaayzxaaWaaeWaaeaaiiGacqWFXoqycqWG0baDdaahaaWcbeqaaiabikdaYiab=j7aIjabgkHiTiabigdaXaaakiabdoeadjabdohaZjabdogaJjabdIgaOnaabmaabaGaemiDaq3aaWbaaSqabeaacqWFYoGyaaaakiaawIcacaGLPaaadaahaaWcbeqaaiabikdaYaaakiabgkHiTiab=f7aHjabdsha0naaCaaaleqabaGae8NSdiMaeyOeI0IaeGymaedaaOGaem4qamKaem4Ba8MaemiDaqNaemiAaG2aaeWaaeaacqWG0baDdaahaaWcbeqaaiab=j7aIbaaaOGaayjkaiaawMcaaaGaayjkaiaawMcaaiabdsfaujabdggaHjabd6gaUjabdIgaOnaadmaabaGaem4vaC1aaeWaaeaacqWG0baDaiaawIcacaGLPaaaaiaawUfacaGLDbaaaeaacqqGMbGzcqqGVbWBcqqGYbGCcqqGGaaicqWG0baDcqGH+aGpcqaIWaamaeaacqaIWaamaeaacqqGMbGzcqqGVbWBcqqGYbGCcqqGGaaicqWG0baDcqGH8aapcqaIWaamaaaacaGL7baaaaa@819D@

where *W*(*t*) = *α*(1 - *t*^*β *^*Coth*(*t*^*β*^))/*β*.

## 3. Hypertabastic survival and hazard Functions

Definition 3.1 (The Hypertabastic Survival Function) Let *T *be a continuous random variable representing the waiting time until the occurrence of an event. Then the hypertabastic survival function is defined as

*S*(*t*) = *P*(*T *> *t*) = *Sech *[*α*(1 - *t*^*β *^*Coth*(*t*^*β*^))/*β*]

where *P *(*T *> *t*) is the probability that waiting time exceeds *t*.

Definition 3.2 (The Hypertabastic Hazard Function) The hypertabastic hazard function *h*(*t*) which represents the instantaneous failure rate at time *t *given survival up to time *t *is defined as

*h*(*t*) = *α*(*t*^2*β*-1^*Csch*(*t*^*β*^)^2 ^- *t*^*β*-1^*Coth*(*t*^*β*^))*Tanh *[*W*(*t*)]

The cumulative hazard function *H*(*t*) is defined as

*H*(*t*) = - ln(*Sech *[*W*(*t*)]).

The hazard function is a conditional failure rate which gives the instantaneous potential for failing at time *t *per unit time for an individual surviving to time *t*.

**Figure 1:**
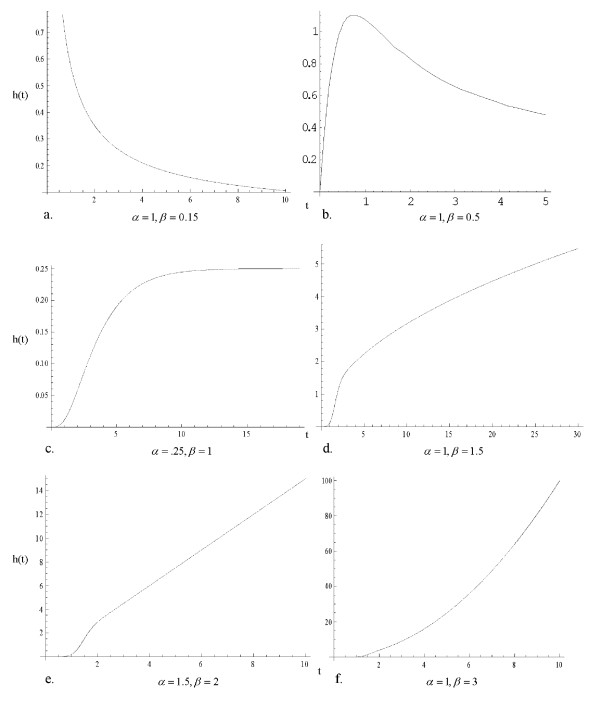
a) Hypertabastic hazard curve for 0 <*β *≤ 0.25; b) Hypertabastic hazard curve for 0.25 <*β *< 1; c) Hypertabastic hazard curve for *β *= 1; d) Hypertabastic hazard curve for 1 ≤ *β *< 2; e) Hypertabastic hazard curve for *β *= 2; f) Hypertabastic hazard curve for *β *> 2.

The most commonly used baseline hazard functions are Weibull, log-normal and log-logistic. The Weibull baseline hazard function has monotone increasing, monotone decreasing and constant forms. The log-normal baseline hazard function is non-monotonic. It follows a behavior that increases to a maximum, and then decreases (unimodal shaped). The log-logistic baseline hazard function can be monotone decreasing or have unimodal shape. These different hazard shapes may reveal different biological mechanisms of disease progression and regression and can provide helpful medical information. The hypertabastic baseline hazard function shapes are given as follows along with illustrative examples from published literature that could fit a pattern of that particular behavior where applicable:

1. The hypertabastic baseline hazard function is monotone decreasing if 0 <*β *≤ 0.25 (Figure 1a). Clark et al. [21] analyzed data for 825 patients diagnosed with primary epithelial ovarian carcinoma and observed that the hazard rate was initially high after the diagnosis and gradually decreased afterwards.

2. The hypertabastic baseline hazard function first increases with time until it reaches its maximum and then decreases (unimodal-shaped) if 0.25 <*β *< 1 (Figure 1b). Demicheli at al. [22] concluded that the hazard rate for node-positive post-menopausal women was unimodal-shaped. Schulman et al. [23] studied the influence of donor and recipient HLA locus mismatching on the development of obliterative bronchiolitis (OB) after lung transplantation and estimated the risk of OB after the transplant. Their estimated hazard function of developing OB indicated a unimodal-shaped curve as well.

3. The hypertabastic baseline hazard initially increases with time, then it reaches its horizontal asymptote *α *provided that *β *= 1 (Figure 1c). Weitz and Fraser [24] concluded that hazard rate plateaus are explained as a generic consequence of considering death in terms of first passage time for processes undergoing a random walk with drift. They analyzed the hazard rate plateau in populations of fruit flies, yeast and other organisms.

4. The hypertabastic baseline hazard function is increasing with upward concavity until it reaches its inflection point and then it continues to increase with downward concavity thereafter if 1 <*β *< 2 (Figure 1d). Such a hazard can be used in many applied sciences when failure rate increases with respect to increase in time. For instance, it can be used to model leukemia survival times for patients not responding to treatment [3].

5. The hypertabastic baseline hazard function is increasing with upward concavity for a while and then it becomes a linear function with slope *α *if *β *= 2. (Figure 1e).

6. The hypertabastic baseline hazard function is increasing with upward concavity if *β *> 2. (Figure 1f).

Definition 3.3 (Disease progression and regression) Let the hazard function be defined on interval *I *and *h'*(*t*) be its derivative.

1. Disease progresses on the time interval *I *if *h'*(*t*) > 0 for all *t *in *I*.

2. Disease regresses on the time interval *I *if *h'*(*t*) < 0 for all *t *in *I*.

3. Disease neither progresses nor regresses on the interval I if *h'*(*t*) = 0 for all *t *in *I*.

Definition 3.4 (Speed of progression and regression) Let *h*(*t*) be a hazard function defined on time interval *I *and let *h'*(*t*) and *h"*(*t*) be first and second derivative of *h*(*t*) respectively.

1. If *h'*(*t*) > 0 and *h"*(*t*) > 0 for all *t *in *I*, then the disease progression speeds up on the time interval *I*.

2. If *h'*(*t*) > 0 and *h"*(*t*) < 0 for all *t *in *I*, then the disease progression slows down on the time interval *I*.

3. If *h'*(*t*) < 0 and *h"*(*t*) > 0 for all *t *in *I*, then the disease regression slows down on the time interval *I*.

4. If *h'*(*t*) < 0 and *h"*(*t*) < 0 for all *t *in *I*, then the disease regression speeds up on the time interval *I*.

Definitions 3.3 and 3.4 can assist clinicians, researches and pharmacologists to monitor disease status over time. If for instance the goal of the drug treatment study is to slow down the disease progression and to understand the time course and management of disease, the above mentioned definitions may be very useful. See for example, the paper by Chan and Holford [25] who studied the rate of deterioration of degenerative diseases over time.

## 4. Hypertabastic proportional hazards model

When the effect of risk factors is to change the baseline hazard function by a proportionate amount at all times *t*, we call the model a proportional hazards model. Suppose vector X is a p-dimensional vector of covariates and assume *g*(*X*|*θ*) is a non negative function of X satisfying the condition that *g*(0|*θ*) = 1. Let *h*_0_(*t*) which has been defined in section 3 as *h*(*t*), be called baseline hazard function. This is the hazard function when there are no covariates in the model. The hypertabastic proportional hazard model assumes a hazard function *h*(*t*|*X*, *θ*) of the form

*h*(*t*|*X*, *θ*) = *h*_0_(*t*)*g*(*X*|*θ*)

where *θ *is a vector of unknown parameters. The hypertabastic survival function *S*(*t*|*X*, *θ*) for the proportional hazards model is defined as

*S*(*t*|*X*, *θ*) = [*S*_0_(*t*)]^*g*(*X*|*θ*)^

where the baseline survival function *S*_0_(*t*) is defined as *S*(*t*) in section 3. The hypertabastic probability density function for the proportional hazard model is denoted by *f*(*t*|*X*, *θ*) and is equal to

*f*(*t*|*X*, *θ*) = *f*_0_(*t*) [*S*_0_(*t*)]^*g*(*X*|*θ*)-1^*g*(*X*|*θ*)

where the baseline probability density function *f*_0_(*t*) is defined as *f*(*t*) in section 2. Depending on the type of censoring, the maximum likelihood function technique along with an appropriate log-likelihood function may be used to estimate the unknown parameters in this model. Most data used in survival analysis have only right censoring, therefore we will focus on right censoring. Consider a sample of right censored survival time's data of n individuals *t*_1_, *t*_2_, ..., *t*_*n *_with associated p-dimensional covariate vectors *X*_1_, *X*_2_, ..., *X*_*n *_and an unknown parameter vector *θ *= (*θ*_1_, *θ*_2_, ..., *θ*_*p*_).

Then, the hypertabastic proportional hazards log-likelihood function can be written as

LL(θ,α,β:X)=∑i=1n(ln⁡[Sech(α(1−tiβCoth(tiβ))/β)]g(Xi|θ)+δiln⁡[((αti−1+2βCsch(tiβ)2−α ti−1+βCoth(tiβ))Tanh(α(1−tiβCoth(tiβ))/β))g(Xi|θ)])
 MathType@MTEF@5@5@+=feaafiart1ev1aaatCvAUfKttLearuWrP9MDH5MBPbIqV92AaeXatLxBI9gBaebbnrfifHhDYfgasaacH8akY=wiFfYdH8Gipec8Eeeu0xXdbba9frFj0=OqFfea0dXdd9vqai=hGuQ8kuc9pgc9s8qqaq=dirpe0xb9q8qiLsFr0=vr0=vr0dc8meaabaqaciaacaGaaeqabaqabeGadaaakeaafaqaaeGabaaabaGaemitaWKaemitaW0aaeWaaeaaiiGacqWF4oqCcqGGSaalcqWFXoqycqGGSaalcqWFYoGycqGG6aGocqWGybawaiaawIcacaGLPaaacqGH9aqpdaaeWbqaaiabcIcaOiGbcYgaSjabc6gaUnaadmaabaGaem4uamLaemyzauMaem4yamMaemiAaG2aaeWaaeaacqWFXoqydaqadaqaaiabigdaXiabgkHiTiabdsha0naaDaaaleaacqWGPbqAaeaacqWFYoGyaaGccqWGdbWqcqWGVbWBcqWG0baDcqWGObaAdaqadaqaaiabdsha0naaDaaaleaacqWGPbqAaeaacqWFYoGyaaaakiaawIcacaGLPaaaaiaawIcacaGLPaaacqGGVaWlcqWFYoGyaiaawIcacaGLPaaaaiaawUfacaGLDbaacqWGNbWzdaqadaqaaiabdIfaynaaBaaaleaacqWGPbqAaeqaaOWaaqqaaeaacqWF4oqCaiaawEa7aaGaayjkaiaawMcaaiabgUcaRaWcbaGaemyAaKMaeyypa0JaeGymaedabaGaemOBa4ganiabggHiLdGccqWF0oazdaWgaaWcbaGaemyAaKgabeaakiGbcYgaSjabc6gaUnaadeaabaWaaeqaaeaadaqabaqaaiab=f7aHjabdsha0naaDaaaleaacqWGPbqAaeaacqGHsislcqaIXaqmcqGHRaWkcqaIYaGmcqWFYoGyaaGccqWGdbWqcqWGZbWCcqWGJbWycqWGObaAdaqadaqaaiabdsha0naaDaaaleaacqWGPbqAaeaacqWFYoGyaaaakiaawIcacaGLPaaadaahaaWcbeqaaiabikdaYaaakiabgkHiTaGaayjkaaaacaGLOaaaaiaawUfaaaqaamaabiaabaWaaeGaaeaacqWFXoqycqqGGaaicqWG0baDdaqhaaWcbaGaemyAaKgabaGaeyOeI0IaeGymaeJaey4kaSIae8NSdigaaOGaem4qamKaem4Ba8MaemiDaqNaemiAaG2aaeWaaeaacqWG0baDdaqhaaWcbaGaemyAaKgabaGae8NSdigaaaGccaGLOaGaayzkaaaacaGLPaaadaqacaqaaiabdsfaujabdggaHjabd6gaUjabdIgaOnaabmaabaGae8xSde2aaeWaaeaacqaIXaqmcqGHsislcqWG0baDdaqhaaWcbaGaemyAaKgabaGae8NSdigaaOGaem4qamKaem4Ba8MaemiDaqNaemiAaG2aaeWaaeaacqWG0baDdaqhaaWcbaGaemyAaKgabaGae8NSdigaaaGccaGLOaGaayzkaaaacaGLOaGaayzkaaGaei4la8Iae8NSdigacaGLOaGaayzkaaaacaGLPaaadaWacaqaaiabdEgaNnaabmaabaGaemiwaG1aaSbaaSqaaiabdMgaPbqabaGcdaabbaqaaiab=H7aXbGaay5bSdaacaGLOaGaayzkaaaacaGLDbaaaiaawMcaaaaaaaa@CE94@

where

δi={0if ti is a right censored observation1otherwise.
 MathType@MTEF@5@5@+=feaafiart1ev1aaatCvAUfKttLearuWrP9MDH5MBPbIqV92AaeXatLxBI9gBaebbnrfifHhDYfgasaacH8akY=wiFfYdH8Gipec8Eeeu0xXdbba9frFj0=OqFfea0dXdd9vqai=hGuQ8kuc9pgc9s8qqaq=dirpe0xb9q8qiLsFr0=vr0=vr0dc8meaabaqaciaacaGaaeqabaqabeGadaaakeaaiiGacqWF0oazdaWgaaWcbaGaemyAaKgabeaakiabg2da9maaceqabaqbaeaabiGaaaqaaiabicdaWaqaaiabbMgaPjabbAgaMjabbccaGiabdsha0naaBaaaleaacqWGPbqAaeqaaOGaeeiiaaIaeeyAaKMaee4CamNaeeiiaaIaeeyyaeMaeeiiaaIaeeOCaiNaeeyAaKMaee4zaCMaeeiAaGMaeeiDaqNaeeiiaaIaee4yamMaeeyzauMaeeOBa4Maee4CamNaee4Ba8MaeeOCaiNaeeyzauMaeeizaqMaeeiiaaIaee4Ba8MaeeOyaiMaee4CamNaeeyzauMaeeOCaiNaeeODayNaeeyyaeMaeeiDaqNaeeyAaKMaee4Ba8MaeeOBa4gabaGaeGymaedabaGaee4Ba8MaeeiDaqNaeeiAaGMaeeyzauMaeeOCaiNaee4DaCNaeeyAaKMaee4CamNaeeyzauMaeiOla4caaaGaay5Eaaaaaa@7097@

Some statistical software packages use logarithm of survival time as their survival time variable in their model fittings. If this is the case, then the following alternative formula can be used as the proportional hazards log-likelihood function:

ALTLL(θ,α,β:X)=∑i=1n(ln⁡[Sech(α(1−tiβCoth(tiβ))/β)]g(Xi|θ)+δiln⁡[ti((αti−1+2βCsch(tiβ)2−α ti−1+βCoth(tiβ))Tanh(α(1−tiβCoth(tiβ))/β))g(Xi|θ)]).
 MathType@MTEF@5@5@+=feaafiart1ev1aaatCvAUfKttLearuWrP9MDH5MBPbIqV92AaeXatLxBI9gBaebbnrfifHhDYfgasaacH8akY=wiFfYdH8Gipec8Eeeu0xXdbba9frFj0=OqFfea0dXdd9vqai=hGuQ8kuc9pgc9s8qqaq=dirpe0xb9q8qiLsFr0=vr0=vr0dc8meaabaqaciaacaGaaeqabaqabeGadaaakeaafaqaaeGabaaabaGaemyqaeKaemitaWKaemivaqLaemitaWKaemitaW0aaeWaaeaaiiGacqWF4oqCcqGGSaalcqWFXoqycqGGSaalcqWFYoGycqGG6aGocqWGybawaiaawIcacaGLPaaacqGH9aqpdaaeWbqaaiabcIcaOiGbcYgaSjabc6gaUnaadmaabaGaem4uamLaemyzauMaem4yamMaemiAaG2aaeWaaeaacqWFXoqydaqadaqaaiabigdaXiabgkHiTiabdsha0naaDaaaleaacqWGPbqAaeaacqWFYoGyaaGccqWGdbWqcqWGVbWBcqWG0baDcqWGObaAdaqadaqaaiabdsha0naaDaaaleaacqWGPbqAaeaacqWFYoGyaaaakiaawIcacaGLPaaaaiaawIcacaGLPaaacqGGVaWlcqWFYoGyaiaawIcacaGLPaaaaiaawUfacaGLDbaacqWGNbWzdaqadaqaaiabdIfaynaaBaaaleaacqWGPbqAaeqaaOWaaqqaaeaacqWF4oqCaiaawEa7aaGaayjkaiaawMcaaiabgUcaRaWcbaGaemyAaKMaeyypa0JaeGymaedabaGaemOBa4ganiabggHiLdGccqWF0oazdaWgaaWcbaGaemyAaKgabeaakiGbcYgaSjabc6gaUnaadeaabaGaemiDaq3aaSbaaSqaaiabdMgaPbqabaGcdaqabaqaamaabeaabaGae8xSdeMaemiDaq3aa0baaSqaaiabdMgaPbqaaiabgkHiTiabigdaXiabgUcaRiabikdaYiab=j7aIbaakiabdoeadjabdohaZjabdogaJjabdIgaOnaabmaabaGaemiDaq3aa0baaSqaaiabdMgaPbqaaiab=j7aIbaaaOGaayjkaiaawMcaamaaCaaaleqabaGaeGOmaidaaOGaeyOeI0cacaGLOaaaaiaawIcaaaGaay5waaaabaWaaeGaaeaadaqacaqaaiab=f7aHjabbccaGiabdsha0naaDaaaleaacqWGPbqAaeaacqGHsislcqaIXaqmcqGHRaWkcqWFYoGyaaGccqWGdbWqcqWGVbWBcqWG0baDcqWGObaAdaqadaqaaiabdsha0naaDaaaleaacqWGPbqAaeaacqWFYoGyaaaakiaawIcacaGLPaaaaiaawMcaamaabiaabaGaemivaqLaemyyaeMaemOBa4MaemiAaG2aaeWaaeaacqWFXoqydaqadaqaaiabigdaXiabgkHiTiabdsha0naaDaaaleaacqWGPbqAaeaacqWFYoGyaaGccqWGdbWqcqWGVbWBcqWG0baDcqWGObaAdaqadaqaaiabdsha0naaDaaaleaacqWGPbqAaeaacqWFYoGyaaaakiaawIcacaGLPaaaaiaawIcacaGLPaaacqGGVaWlcqWFYoGyaiaawIcacaGLPaaaaiaawMcaamaadiaabaGaem4zaC2aaeWaaeaacqWGybawdaWgaaWcbaGaemyAaKgabeaakmaaeeaabaGae8hUdehacaGLhWoaaiaawIcacaGLPaaaaiaaw2faaaGaayzkaaGaeiOla4caaaaa@D5D7@

The maximum likelihood estimate of (p+2) dimensional parameter vector *λ *= (*α*, *β*, *θ*_1_, *θ*_2_, ..., *θ*_*p*_) is the vector λ^=(α^,β^,θ^1,θ^2,...,θ^p)
 MathType@MTEF@5@5@+=feaafiart1ev1aaatCvAUfKttLearuWrP9MDH5MBPbIqV92AaeXatLxBI9gBaebbnrfifHhDYfgasaacH8akY=wiFfYdH8Gipec8Eeeu0xXdbba9frFj0=OqFfea0dXdd9vqai=hGuQ8kuc9pgc9s8qqaq=dirpe0xb9q8qiLsFr0=vr0=vr0dc8meaabaqaciaacaGaaeqabaqabeGadaaakeaaiiGacuWF7oaBgaqcaiabg2da9iabcIcaOiqb=f7aHzaajaGaeiilaWIaf8NSdiMbaKaacqGGSaalcuWF4oqCgaqcamaaBaaaleaacqaIXaqmaeqaaOGaeiilaWIaf8hUdeNbaKaadaWgaaWcbaGaeGOmaidabeaakiabcYcaSiabc6caUiabc6caUiabc6caUiabcYcaSiqb=H7aXzaajaGaemiCaaNaeiykaKcaaa@448B@. Asymptotically, λ^
 MathType@MTEF@5@5@+=feaafiart1ev1aaatCvAUfKttLearuWrP9MDH5MBPbIqV92AaeXatLxBI9gBaebbnrfifHhDYfgasaacH8akY=wiFfYdH8Gipec8Eeeu0xXdbba9frFj0=OqFfea0dXdd9vqai=hGuQ8kuc9pgc9s8qqaq=dirpe0xb9q8qiLsFr0=vr0=vr0dc8meaabaqaciaacaGaaeqabaqabeGadaaakeaaiiGacuWF7oaBgaqcaaaa@2E77@ is normally distributed with mean vector *λ *and variance-covariance matrix V. An estimate of V can be obtained by calculating the inverse of the observed information matrix.

To assess the statistical significance of model parameters, one can use the well known statistical tests such as likelihood ratio test, Wald test, or the score test.

The hazard ratio *HR*(*X*_*i*_, *X*_*j*_) for individuals *i *and *j *with covariate vectors *X*_*i *_and *X*_*j *_is given by

*HR*(*X*_*i*_, *X*_*j*_) = *g*(*X*_*i*_|*θ*)/*g*(*X*_*j*_|*θ*).

The most common form of *g*(*X*|*θ*) is exp [-*θ*^*T *^*X*] [1]. If we use g(X|θ)=exp⁡[−θTX]=exp⁡(−∑k=1pθkxk)
 MathType@MTEF@5@5@+=feaafiart1ev1aaatCvAUfKttLearuWrP9MDH5MBPbIqV92AaeXatLxBI9gBaebbnrfifHhDYfgasaacH8akY=wiFfYdH8Gipec8Eeeu0xXdbba9frFj0=OqFfea0dXdd9vqai=hGuQ8kuc9pgc9s8qqaq=dirpe0xb9q8qiLsFr0=vr0=vr0dc8meaabaqaciaacaGaaeqabaqabeGadaaakeaacqWGNbWzdaqadaqaaiabdIfaynaaeeaabaacciGae8hUdehacaGLhWoaaiaawIcacaGLPaaacqGH9aqpcyGGLbqzcqGG4baEcqGGWbaCdaWadaqaaiabgkHiTiab=H7aXnaaCaaaleqabaGaemivaqfaaOGaemiwaGfacaGLBbGaayzxaaGaeyypa0JagiyzauMaeiiEaGNaeiiCaa3aaeWaaeaacqGHsisldaaeWbqaaiab=H7aXnaaBaaaleaacqWGRbWAaeqaaOGaemiEaG3aaSbaaSqaaiabdUgaRbqabaaabaGaem4AaSMaeyypa0JaeGymaedabaGaemiCaahaniabggHiLdaakiaawIcacaGLPaaaaaa@557A@, then the hazard ratio *HR*(*X*_*i*_|*X*_*j*_) = exp [-*θ*^*T *^(*X*_*i *_- *X*_*j*_)] where *HR*(*X*_*i*_, *X*_*j*_) is independent of time *t*. If *X*_*i *_= *X *and *X*_*j *_= 0, then the hazard ratio becomes *HR*(*X*_*i*_, 0) = *g*(*X*|*θ*) and if we use g(X|θ)=exp⁡(−∑k=1pθkxk)
 MathType@MTEF@5@5@+=feaafiart1ev1aaatCvAUfKttLearuWrP9MDH5MBPbIqV92AaeXatLxBI9gBaebbnrfifHhDYfgasaacH8akY=wiFfYdH8Gipec8Eeeu0xXdbba9frFj0=OqFfea0dXdd9vqai=hGuQ8kuc9pgc9s8qqaq=dirpe0xb9q8qiLsFr0=vr0=vr0dc8meaabaqaciaacaGaaeqabaqabeGadaaakeaacqWGNbWzdaqadaqaaiabdIfaynaaeeaabaacciGae8hUdehacaGLhWoaaiaawIcacaGLPaaacqGH9aqpcyGGLbqzcqGG4baEcqGGWbaCdaqadaqaaiabgkHiTmaaqahabaGae8hUde3aaSbaaSqaaiabdUgaRbqabaGccqWG4baEdaWgaaWcbaGaem4AaSgabeaaaeaacqWGRbWAcqGH9aqpcqaIXaqmaeaacqWGWbaCa0GaeyyeIuoaaOGaayjkaiaawMcaaaaa@490F@, then ln⁡[g(X|θ)]=−∑k=1pθkxk
 MathType@MTEF@5@5@+=feaafiart1ev1aaatCvAUfKttLearuWrP9MDH5MBPbIqV92AaeXatLxBI9gBaebbnrfifHhDYfgasaacH8akY=wiFfYdH8Gipec8Eeeu0xXdbba9frFj0=OqFfea0dXdd9vqai=hGuQ8kuc9pgc9s8qqaq=dirpe0xb9q8qiLsFr0=vr0=vr0dc8meaabaqaciaacaGaaeqabaqabeGadaaakeaacyGGSbaBcqGGUbGBdaWadaqaaiabdEgaNnaabmaabaGaemiwaG1aaqqaaeaaiiGacqWF4oqCaiaawEa7aaGaayjkaiaawMcaaaGaay5waiaaw2faaiabg2da9iabgkHiTmaaqahabaGae8hUde3aaSbaaSqaaiabdUgaRbqabaGccqWG4baEdaWgaaWcbaGaem4AaSgabeaaaeaacqWGRbWAcqGH9aqpcqaIXaqmaeaacqWGWbaCa0GaeyyeIuoaaaa@4800@, where -*θ*_*j*_*x*_*j *_is the elasticity of hazard rate with respect to covariate *x*_*j*_. Symbolically,

[∂*h*(*t*|*X*, *θ*)/∂*x*_*j*_]·[*x*_*j*_/*h*(*t*|*X*, *θ*)] = -*θ *_*j*_*x*_*j*_.

The elasticity of hazard function with respect to covariate *x*_*j *_is a measure of the responsiveness of the hazard function to change in covariate *x*_*j*_. Intuitively, elasticity of the hazard function with respect to change in covariate *x*_*j *_is percent change in failure rate divided by percent change in covariate *x*_*j*_.

## 5. Hypertabastic accelerated failure time model

When the covariates act multiplicatively on the time-scale, the model is called accelerated failure time model [3,4]. The hypertabastic accelerated failure time model assumes a hazard function *h*(*t*|*X*, *θ*) of the form

*h*(*t*|*X*, *θ*) = *h*_0_(*tg*|*X*, *θ*))*g*(*X*, *θ*)

where *h*_0_(•) is the baseline hypertabastic hazard function and the hypertabastic survival function for the accelerated failure time model is

*S*(*t*|*X*, *θ*) = *S*_0_(*tg*(*X*|*θ*))

where *S*_0_(•) is the baseline hypertabastic survival function. Finally, the hypertabastic probability density function for the accelerated failure time model is

*f*(*t*|*X*, *θ*) = *f*_0_(*tg*(*X*|*θ*))*g*(*X*|*θ*)

where *f*_0_(•) is the baseline hypertabastic probability density function. The maximum likelihood technique can be used to estimate the parameters of this model and statistical tests similar to section 4 can be used to assess the significance of model covariates. For the right censored data, the hypertabastic accelerated failure time model would have a log-likelihood function of the form

LL(θ,α,β:X)=∑i=1n(ln⁡[Sech(α(1−(Z(ti))βCoth((Z(ti))β))/β)]+δiln⁡[((α(Z(ti))−1+2βCsch[(Z(ti))β]2−α(Z(ti))−1+βCoth((Z(ti))β))Tanh(α(1−(Z(ti))βCoth((Z(ti))β))/β))g(Xi|θ)])
 MathType@MTEF@5@5@+=feaafiart1ev1aaatCvAUfKttLearuWrP9MDH5MBPbIqV92AaeXatLxBI9gBaebbnrfifHhDYfgasaacH8akY=wiFfYdH8Gipec8Eeeu0xXdbba9frFj0=OqFfea0dXdd9vqai=hGuQ8kuc9pgc9s8qqaq=dirpe0xb9q8qiLsFr0=vr0=vr0dc8meaabaqaciaacaGaaeqabaqabeGadaaakeaafaqaaeGabaaabaGaemitaWKaemitaW0aaeWaaeaaiiGacqWF4oqCcqGGSaalcqWFXoqycqGGSaalcqWFYoGycqGG6aGocqWGybawaiaawIcacaGLPaaacqGH9aqpdaaeWbqaaiabcIcaOiGbcYgaSjabc6gaUnaadmaabaGaem4uamLaemyzauMaem4yamMaemiAaG2aaeWaaeaacqWFXoqycqGGOaakcqaIXaqmcqGHsisldaqadaqaaiabdQfaAnaabmaabaGaemiDaq3aaSbaaSqaaiabdMgaPbqabaaakiaawIcacaGLPaaaaiaawIcacaGLPaaadaahaaWcbeqaaiab=j7aIbaakiabdoeadjabd+gaVjabdsha0jabdIgaOnaabmaabaWaaeWaaeaacqWGAbGwdaqadaqaaiabdsha0naaBaaaleaacqWGPbqAaeqaaaGccaGLOaGaayzkaaaacaGLOaGaayzkaaWaaWbaaSqabeaacqWFYoGyaaaakiaawIcacaGLPaaacqGGPaqkcqGGVaWlcqWFYoGyaiaawIcacaGLPaaaaiaawUfacaGLDbaacqGHRaWkaSqaaiabdMgaPjabg2da9iabigdaXaqaaiabd6gaUbqdcqGHris5aOGae8hTdq2aaSbaaSqaaiabdMgaPbqabaGccyGGSbaBcqGGUbGBdaWabaqaamaabeaabaWaaeqaaeaacqWFXoqydaqadaqaaiabdQfaAnaabmaabaGaemiDaq3aaSbaaSqaaiabdMgaPbqabaaakiaawIcacaGLPaaaaiaawIcacaGLPaaadaahaaWcbeqaaiabgkHiTiabigdaXiabgUcaRiabikdaYiab=j7aIbaaaOGaayjkaaaacaGLOaaaaiaawUfaaaqaamaabiaabaWaaeGaaeaacqWGdbWqcqWGZbWCcqWGJbWycqWGObaAdaWadaqaamaabmaabaGaemOwaO1aaeWaaeaacqWG0baDdaWgaaWcbaGaemyAaKgabeaaaOGaayjkaiaawMcaaaGaayjkaiaawMcaamaaCaaaleqabaGae8NSdigaaaGccaGLBbGaayzxaaWaaWbaaSqabeaacqaIYaGmaaGccqGHsislcqWFXoqydaqadaqaaiabdQfaAnaabmaabaGaemiDaq3aaSbaaSqaaiabdMgaPbqabaaakiaawIcacaGLPaaaaiaawIcacaGLPaaadaahaaWcbeqaaiabgkHiTiabigdaXiabgUcaRiab=j7aIbaakiabdoeadjabd+gaVjabdsha0jabdIgaOnaabmaabaWaaeWaaeaacqWGAbGwdaqadaqaaiabdsha0naaBaaaleaacqWGPbqAaeqaaaGccaGLOaGaayzkaaaacaGLOaGaayzkaaWaaWbaaSqabeaacqWFYoGyaaaakiaawIcacaGLPaaaaiaawMcaamaabiaabaGaemivaqLaemyyaeMaemOBa4MaemiAaG2aaeWaaeaacqWFXoqycqGGOaakcqaIXaqmcqGHsisldaqadaqaaiabdQfaAnaabmaabaGaemiDaq3aaSbaaSqaaiabdMgaPbqabaaakiaawIcacaGLPaaaaiaawIcacaGLPaaadaahaaWcbeqaaiab=j7aIbaakiabdoeadjabd+gaVjabdsha0jabdIgaOnaabmaabaWaaeWaaeaacqWGAbGwdaqadaqaaiabdsha0naaBaaaleaacqWGPbqAaeqaaaGccaGLOaGaayzkaaaacaGLOaGaayzkaaWaaWbaaSqabeaacqWFYoGyaaaakiaawIcacaGLPaaacqGGPaqkcqGGVaWlcqWFYoGyaiaawIcacaGLPaaaaiaawMcaamaadiaabaGaem4zaC2aaeWaaeaacqWGybawdaWgaaWcbaGaemyAaKgabeaakmaaeeaabaGae8hUdehacaGLhWoaaiaawIcacaGLPaaaaiaaw2faaaGaayzkaaaaaaaa@E9C1@

where *Z*(*t*_*i*_) = *t*_*i*_*g*(*X*_*i*_|*θ*).

Again, if someone prefers to use the logarithm of time as the model survival time variable, then the alternative accelerated failure time log-likelihood function is

ALTLL(θ,α,β:X)=∑i=1n(ln⁡[Sech(α(1−(Z(ti))βCoth((Z(ti))β))/β)]+δiln⁡[ti((α(Z(ti))−1+2βCsch[(Z(ti))β]2−α(Z(ti))−1+βCoth((Z(ti))β))Tanh(α(1−(Z(ti))βCoth((Z(ti))β))/β))g(Xi|θ)]).
 MathType@MTEF@5@5@+=feaafiart1ev1aaatCvAUfKttLearuWrP9MDH5MBPbIqV92AaeXatLxBI9gBaebbnrfifHhDYfgasaacH8akY=wiFfYdH8Gipec8Eeeu0xXdbba9frFj0=OqFfea0dXdd9vqai=hGuQ8kuc9pgc9s8qqaq=dirpe0xb9q8qiLsFr0=vr0=vr0dc8meaabaqaciaacaGaaeqabaqabeGadaaakeaafaqaaeGabaaabaGaemyqaeKaemitaWKaemivaqLaemitaWKaemitaW0aaeWaaeaaiiGacqWF4oqCcqGGSaalcqWFXoqycqGGSaalcqWFYoGycqGG6aGocqWGybawaiaawIcacaGLPaaacqGH9aqpdaaeWbqaaiabcIcaOiGbcYgaSjabc6gaUnaadmaabaGaem4uamLaemyzauMaem4yamMaemiAaG2aaeWaaeaacqWFXoqycqGGOaakcqaIXaqmcqGHsisldaqadaqaaiabdQfaAnaabmaabaGaemiDaq3aaSbaaSqaaiabdMgaPbqabaaakiaawIcacaGLPaaaaiaawIcacaGLPaaadaahaaWcbeqaaiab=j7aIbaakiabdoeadjabd+gaVjabdsha0jabdIgaOnaabmaabaWaaeWaaeaacqWGAbGwdaqadaqaaiabdsha0naaBaaaleaacqWGPbqAaeqaaaGccaGLOaGaayzkaaaacaGLOaGaayzkaaWaaWbaaSqabeaacqWFYoGyaaaakiaawIcacaGLPaaacqGGPaqkcqGGVaWlcqWFYoGyaiaawIcacaGLPaaaaiaawUfacaGLDbaacqGHRaWkaSqaaiabdMgaPjabg2da9iabigdaXaqaaiabd6gaUbqdcqGHris5aOGae8hTdq2aaSbaaSqaaiabdMgaPbqabaGccyGGSbaBcqGGUbGBdaWabaqaaiabdsha0naaBaaaleaacqWGPbqAaeqaaOWaaeqaaeaadaqabaqaaiab=f7aHnaabmaabaGaemOwaO1aaeWaaeaacqWG0baDdaWgaaWcbaGaemyAaKgabeaaaOGaayjkaiaawMcaaaGaayjkaiaawMcaamaaCaaaleqabaGaeyOeI0IaeGymaeJaey4kaSIaeGOmaiJae8NSdigaaaGccaGLOaaaaiaawIcaaaGaay5waaaabaWaaeGaaeaadaqacaqaaiabdoeadjabdohaZjabdogaJjabdIgaOnaadmaabaWaaeWaaeaacqWGAbGwdaqadaqaaiabdsha0naaBaaaleaacqWGPbqAaeqaaaGccaGLOaGaayzkaaaacaGLOaGaayzkaaWaaWbaaSqabeaacqWFYoGyaaaakiaawUfacaGLDbaadaahaaWcbeqaaiabikdaYaaakiabgkHiTiab=f7aHnaabmaabaGaemOwaO1aaeWaaeaacqWG0baDdaWgaaWcbaGaemyAaKgabeaaaOGaayjkaiaawMcaaaGaayjkaiaawMcaamaaCaaaleqabaGaeyOeI0IaeGymaeJaey4kaSIae8NSdigaaOGaem4qamKaem4Ba8MaemiDaqNaemiAaG2aaeWaaeaadaqadaqaaiabdQfaAnaabmaabaGaemiDaq3aaSbaaSqaaiabdMgaPbqabaaakiaawIcacaGLPaaaaiaawIcacaGLPaaadaahaaWcbeqaaiab=j7aIbaaaOGaayjkaiaawMcaaaGaayzkaaWaaeGaaeaacqWGubavcqWGHbqycqWGUbGBcqWGObaAdaqadaqaaiab=f7aHjabcIcaOiabigdaXiabgkHiTmaabmaabaGaemOwaO1aaeWaaeaacqWG0baDdaWgaaWcbaGaemyAaKgabeaaaOGaayjkaiaawMcaaaGaayjkaiaawMcaamaaCaaaleqabaGae8NSdigaaOGaem4qamKaem4Ba8MaemiDaqNaemiAaG2aaeWaaeaadaqadaqaaiabdQfaAnaabmaabaGaemiDaq3aaSbaaSqaaiabdMgaPbqabaaakiaawIcacaGLPaaaaiaawIcacaGLPaaadaahaaWcbeqaaiab=j7aIbaaaOGaayjkaiaawMcaaiabcMcaPiabc+caViab=j7aIbGaayjkaiaawMcaaaGaayzkaaWaamGaaeaacqWGNbWzdaqadaqaaiabdIfaynaaBaaaleaacqWGPbqAaeqaaOWaaqqaaeaacqWF4oqCaiaawEa7aaGaayjkaiaawMcaaaGaayzxaaaacaGLPaaacqGGUaGlaaaaaa@F104@

The maximum likelihood estimate of (p+2) dimensional parameter vector (*α*, *β*, *θ*_1_, *θ*_2_, ..., *θ*_*p*_) and testing of hypothesis regarding model parameters are similar to methods of section 4.

## 6. Hypertabastic proportional odds model

If the effect of risk factor is to change the odds of survival beyond time *t *by a proportionate amount, then the model is called proportional odds model. The odds of surviving beyond time *t *are expressed as

*S*(*t*|*X*, *θ*)/(1 - *S*(*t*|*X*, *θ*)) = *g*(*X*|*θ*)*S*_0_(*t*)/(1 - *S*_0_(*t*))

where *S*_0_(*t*) is the baseline hypertabastic survival function. The hypertabastic survival function for the proportional odds model is given by

*S*(*t*|*X*, *θ*) = 1/[1+((1 - *S*_0_(*t*))/*S*_0_(*t*))*g*(*X*|*θ*)]

and the hypertabastic baseline odds function of survival beyond time *t *is given by

*S*_0_(*t*)/(1 - *S*_0_(*t*)) = *Sech *[*W*(*t*)]/(1 - *Sech *[*W*(*t*)]).

The ratio of the odds of survival of individual *i *relative to individual *j *is equal to exp [-*θ*^*T*^(*X*_*i *_- *X*_*j*_)]. The hypertabastic proportional odds model can be fitted by maximizing an appropriate likelihood function.

## 7. Simulation study

To evaluate the performance of the hypertabastic model we conduct a simulation study in which we compare the overall fit of the proposed model with Weibull, log-logistic and log-normal models. Since all distributions under our consideration have exactly two parameters, we will use the negative of the log-likelihood as a measure of goodness-of-fit. This measure would result in the same conclusion as the Akaike's Information Criterion (AIC) [26]. Thus the smallest value suggests a better fit. We conduct 1000 simulations with sample size of 200 and random censoring of approximately 40%. We sample time-to-event from 11 different parameter combinations of two parameter Weibull, log-normal and gamma distributions for a total of 33 combinations or 33000 simulations. We fit four mentioned models and average the -log likelihood over 1000 runs to determine which model fits the simulated data with the overall most precision on the average. Simulation results are presented in Table 1.

**Table 1:** Average -log likelihoods and their standard errors for Weibull (W), hypertabastic (HT), log-logistic (LL) and log-normal (LN) models based on 1000 simulations from eleven parameter combinations of two parameter Weibull, log-normal and gamma distributions

	W(1,1)	W(1,2)	W(2,1)	W(1,3)	W(3,1)	W(1,4)	W(4,1)	W(.5,.5)	W(.5,1)	W(1,.5)	W(2,3)
W	180.2	263.4	132	312.2	94.5	347.1	66.2	110.3	192.8	97.5	263.6
	(8.8)	(12.1)	(6.8)	(14.1)	(8.6)	(16.2)	(9.2)	(21.4)	(21)	(8.6)	(10.7)
HT	181.9	265.4	133.7	314.5	96.1	349.6	67.8	112.2	194.6	98.9	267
	(9.1)	(12.4)	(7.1)	(14.5)	(8.9)	(16.6)	(9.3)	(21.3)	(21.1)	(8.6)	(11.2)
LL	183.8	267.1	135.7	316	98.1	350.9	69.8	114	196.1	101.1	267.4
	(9.3)	(12.7)	(7.3)	(14.8)	(9)	(16.9)	(9.3)	(21.5)	(21.3)	(8.8)	(11.4)
LN	188.3	271.6	140	320.4	102.5	355.3	74	118.4	200.8	105.3	272
	(10.4)	(13.4)	(8.6)	(15.7)	(8.1)	(17.3)	(10.3)	(21.5)	(21.7)	(9.7)	(11.4)

											
	LN(1,1)	LN(1,2)	LN(2,1)	LN(1,3)	LN(3,1)	LN(1,4)	LN(4,1)	LN(.5,.5)	LN(.5,1)	LN(1,.5)	LN(2,3)

W	359.7	443.7	478.6	494.2	600.2	530.1	719.5	216.8	300	277	610.3
	(18.6)	(27)	(23.6)	(37)	(30.4)	(90.8)	(34.7)	(11.2)	(15.5)	(12.4)	(41)
HT	352.2	436.7	470.4	486.6	591.7	517.4	712	209.3	292.9	268.7	603.2
	(17.6)	(26.5)	(23.1)	(35)	(29.7)	(46.4)	(34.1)	(10.3)	(14.9)	(11.7)	(40.6)
LL	351.9	436.1	470.8	486.7	592.4	516.5	711.8	209	292.3	269.1	602.6
	(17.6)	(26.6)	(23.1)	(36.9)	(29.7)	(46.6)	(34.2)	(10.2)	(15)	(11.7)	(40.7)
LN	350.4	434.3	469.4	485.2	590.9	515	710.4	207.6	290.9	267.7	601.5
	(17.6)	(26.6)	(23.1)	(36.9)	(29.6)	(46.6)	(34.2)	(10.1)	(14.9)	(11.6)	(40.5)

											
	G(1,1)	G(1,2)	G(2,1)	G(1,3)	G(3,1)	G(4,1)	G(4,1)	G(.5,.5)	G(.5,1)	G(1,.5)	G(2,3)

W	180.3	97.4	250.8	48.7	283.7	305.5	305.6	155	71.9	263.2	118.3
	(8.8)	(8.5)	(10)	(9.2)	(12.1)	(13.3)	(13.3)	(15.7)	(15.4)	(12.1)	(7.6)
HT	181.9	98.9	250.5	50.2	282.6	303.5	303.6	159.2	75.8	265.1	117.8
	(9.1)	(8.7)	(10.1)	(9.3)	(12.2)	(13.2)	(13.2)	(15.8)	(15.5)	(12.5)	(7.7)
LL	183.9	101.1	251.5	52.4	283.1	303.9	303.9	162.1	78.9	266.8	119.1
	(9.3)	(8.9)	(10.3)	(9.3)	(12.3)	(13.2)	(13.3)	(15.9)	(15.5)	(12.8)	(7.8)
LN	188.3	105.6	253.6	57.1	284.2	304.5	304.6	169.6	86.4	271.1	121.3
	(10.3)	(9.8)	(10.8)	(10.2)	(12.7)	(13.4)	(13.4)	(16.4)	(16.2)	(13.7)	(8.6)

In simulations where we sample from a two-parameter Weibull distribution, obviously, the Weibull model fits the data with highest precision in all instances. Hypertabastic model is a close second, outperforming log-normal and log-logistic models for all combinations of parameters, with log-normal being the worst. Similarly when sampling is from a two-parameter log-normal distribution, the log-normal model outperforms all other models. The hypertabastic model and the log-logistic show similar results, with the log-logistic being slightly better in eight combinations of parameters and the Weibull model performing the worst. Finally, when sampling from a two parameter gamma distribution, the Weibull model fits with the most precision in seven out of eleven combinations. That is because the Weibull distribution and the gamma distribution have hazard functions which are similar in shapes. In another four instances, the hypertabastic model slightly outperforms the Weibull model. The log-logistic model comes in third, however it is close to the hypertabastic and the Weibull for several combinations, while the log-normal does the worst in all instances.

## 8. Application

All models presented in the next two examples were fitted using Mathematica 6.0 (Wolfram Research, Inc.) and SASv9 (SAS Institute Inc., Cary, NC). They were fitted using both *time *and *log(time)*. However, only *log(time) *results are presented for the multiple myeloma proportional hazards and brain cancer accelerated failure time models since *log(time) *is commonly used as a default approach in many packages and procedures. Besides the hypertabastic survival model we fit Weibull, log-normal, log-logistic and Cox models. We present all parameter estimates along with their standard errors and compare -2log-likelihood estimates, as well as AIC, to assess the goodness-of-fit of the models. SAS programs used to fit the hypertabastic proportional hazards models with *time *and *log(time) *for multiple myeloma data are provided [see Additional file 1].

### 8.1 Analysis of multiple myeloma data

**Figure 2:**
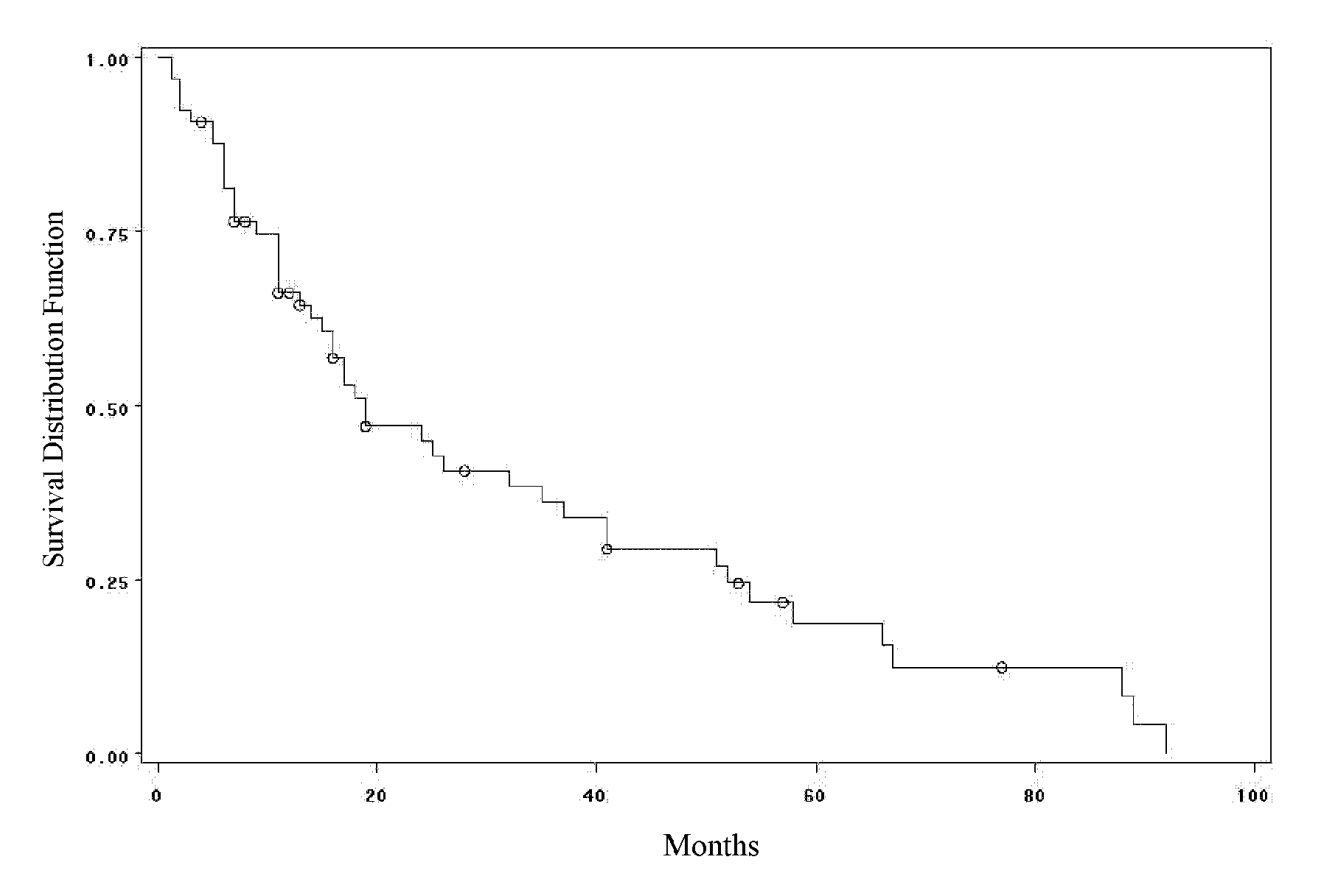
Kaplan-Meier survival curve for multiple myeloma.

**Figure 3:**
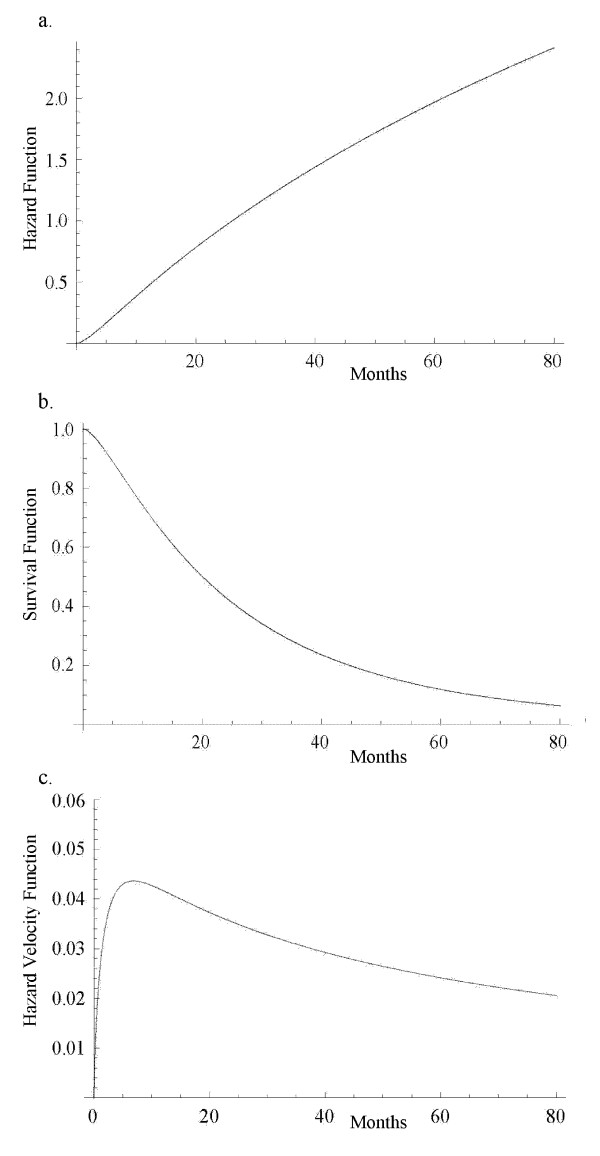
a) Hypertabastic hazard curve for multiple myeloma; b) Hypertabastic survival curve for multiple myeloma; c) Hypertabastic velocity of hazard curve for multiple myeloma.

**Figure 4:**
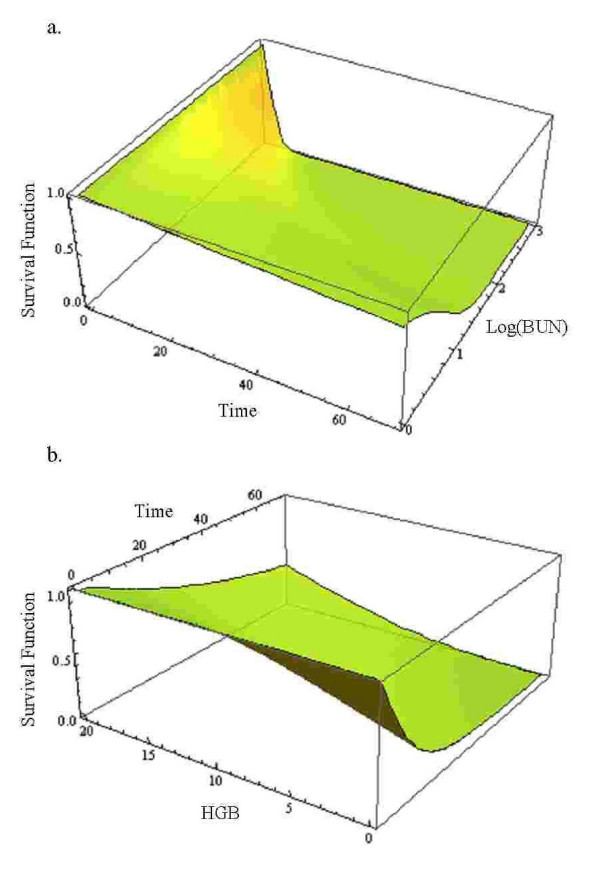
a) Hypertabastic 3D survival curve with variables time and Log (BUN) for multiple myeloma data; b) Hypertabastic 3D survival curve with variables time and HGB for multiple myeloma data.

Multiple myeloma is a cancer formed by abnormal white blood cells, called malignant plasma cells. A malignant monoclonal plasma cell is called plasmacytoma. If the disease spreads throughout multiple bone marrow sites in the body, it is called multiple myeloma. This disease weakens a patient's immune system and is usually difficult to cure. To investigate the performance of the hypertabastic proportional hazards model and to compare it with Cox, Weibull, log-logistic and log-normal models, we analyze a cancer data set obtained from a study conducted by Krall et al. [27]. The data contains information on 65 patients with multiple myeloma in which the patients were treated with alkylating agents. This drug is designed to interfere with the cell's DNA and inhibits cancer cell growth. Of these 65 patients, only 17 survived the duration of the study. The data is right-censored and the survival time from the date of diagnosis is measured in months. The covariates have been measured at diagnosis and the covariate list consists of a logarithm of white blood cell count, serum calcium, presence or absence of Bence Jones protein, proteinuria, gender, age, percent myeloid cells in peripheral blood, percent lymphocytes in peripheral blood, logarithm of percent plasma cells in bone marrow, total serum protein, presence or absence of infection, serum globin, logarithm of blood urea nitrogen, fractures, platelets, and hemoglobin. Our first task is to select risk factors that are statistically significant. To accomplish this task we use the hypertabastic log-likelihood function for proportional hazards model and follow the general variable selection strategy outlined by Collet [4]. We also examine the possibility of interactions. The two most significant risk factors that we found are the logarithm of blood urea nitrogen and hemoglobin. Using the stepwise regression, we fit the Cox proportional hazards model. The Kaplan-Meier survival curve for multiple myeloma data is shown in figure 2. The Cox regression did identify the same variables as the most significant prognostic factors. Table 2 gives results for the hypertabastic and the four comparison models. It shows that the -2log-likelihood and AIC statistics are lowest for the hypertabastic model when fitted to the multiple myeloma data. This indicates that the hypertabastic proportional hazards model fits the multiple myeloma data best. The most significant single variable identified by the hypertabastic model is the logarithm of blood urea nitrogen with an estimated chi-square value of 11.11 (p = 0.0014). The second significant variable is hemoglobin. All other models have identified these two variables as the most significant ones. The mean levels of patients' hemoglobin and the logarithm of blood urea nitrogen are 10.20 and 1.39 respectively. At this level, the median survival time for the hypertabastic, log-normal, log-logistic, Weibull and Cox are 20.04, 19.29, 21.01, 21.92 and 19 months respectively. Figures 3a and 3b show the graph of hypertabastic hazard and survival functions for multiple myeloma data. Figure 3a clearly shows that the hypertabastic hazard function is an increasing function of time. By examining figure 3b, we realize that for patients with a Log (BUN) reading of 1.39 and a hemoglobin level of 10.20 there is about a10% chance of survival beyond 65 months. At the above mentioned mean levels for the hemoglobin and logarithm of blood urea nitrogen, the hypertabastic hazard function shows that the failure rate (hazard) reaches its maximum velocity in about 5.15 months. At this point the disease progression has its highest speed (Figure 3c). Figures 4a and 4b show the 3-dimensional graphs for survival of multiple myeloma patients as functions of time and Log(BUN), as well as time and HGB. Other models under consideration had monotone increasing hazard functions (graphs not shown).

**Table 2:** Statistical results for hypertabastic proportional hazards and four comparison models for multiple myeloma data

	-2Log likelihood	AIC	Estimate	SE	Chi-square	p-value
Hypertabastic	162.33	170.33				
*α*			0.0853	0.0438	3.79	0.0559
*β*			0.4349	0.0712	37.34	<0.0001
Log(BUN)			1.8986	0.5697	11.11	0.0014
Hemogolobin			-0.0974	0.0539	3.27	0.0752
Weibull	162.66	170.66				
*α*			0.0056	0.0067	0.70	0.4041
*β*			1.1403	0.1229	86.08	<0.0001
Log(BUN)			1.7451	0.5999	8.46	0.0049
Hemogolobin			-0.1112	0.0561	3.93	0.0517
Log-normal	162.61	170.61				
*α*			0.0078	0.0095	0.67	0.4145
*β*			1.6153	0.3089	27.35	<0.0001
Log(BUN)			1.8935	0.5703	11.02	0.0015
Hemogolobin			-0.0929	0.0536	3.01	0.0876
Log-logistic	163.10	171.10				
*α*			-5.6519	0.9369	36.39	<0.0001
*β*			1.2401	0.1452	72.96	<0.0001
Log(BUN)			1.8651	0.5464	11.65	0.0011
Hemogolobin			-0.0997	0.0526	3.58	0.0628
Cox	296.08	302.08				
Log(BUN)			1.6744	0.6121	7.48	0.0062
Hemogolobin			-0.1189	0.0575	4.28	0.0385

### 8.2 Analysis of glioma brain cancer data

**Figure 5:**
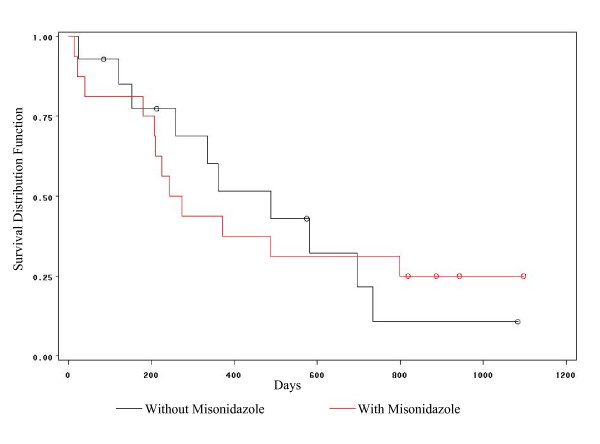
Kaplan-Meier survival curves for glioma brain cancer.

Glioma is a cancer of the brain which begins in glial cells. These cells support the neurons. These cells have a very high rate of growth which can quickly destroy the normal cells. The primary types of glioma cancers are astrocytomas, ependymomas and oligodendrogliomas. Shin et al. [28] studied ways to improve the effectiveness of radiation in the treatment of cerebral malignant astrocytoma. Their study focused on the assessment of the effect of multiple daily fractionated radiation therapy with and without misonidazole. They concluded that the addition of misonidazole did not significantly improve the patients' survival. In this section we apply the hypertabastic accelerated failure time technique to model the survival time of a sample of 30 patients from the randomized trials of radiotherapy with and without the radiosensitizer misinidazole. The data was obtained from the Medical Research Council Working Party (MRC) on misonidazole in gliomas. This data is right censored and has been previously analyzed for the selection of variables by MRC [29]. Survival time was measured in days and the longest survival time was 1098 days. We compared radiotherapy treatment of brain cancer patients with radiosensitizer misonidazole to radiotherapy without misonidazole. Figure 5 shows a Kaplan-Meier plot of the estimated survival curves for both groups. The log-rank, Wilcoxon, and likelihood ratio tests are all non-significant, suggesting no significant difference between survival curves for the two groups. The Kaplan-Meier estimates of median survival time for radiotherapy with misonidazole group is 258.5 days and for the radiotherapy without misonidazole group is 488 days. The overall median survival time for both groups combined is 361 days. Using the Kaplan-Meier estimates of survival function, a plot of log-cumulative hazard function against the logarithm of the survival time for individuals in two groups indicates that the data is coming from a Weibul distribution. Knowing this information led us to evaluate the performance of the hypertabastic model. First, we fit a hypertabastic accelerated failure time model to analyze the brain cancer data. Then the hypertabastic accelerated failure time model is compared with Weibul, log-logistic, log-normal accelerated failure time models and the Cox proportional hazards model. This model incorporates a binary covariate coded as treatment = 1 for the type of radiotherapy with misonidazole, and as treatment = 0 for radiotherapy without misonidazole. The second covariate is the age of the patient. Thus, this model contains two covariates- treatment and age. We use the method of maximum likelihood to maximize hypertabastic accelerated failure time log-likelihood function for right censored data discussed in section 5. Table 3 gives a statistical summary for the glioma data. For instance, the hypertabastic estimated value for the coefficient of the variable radiosensitizer is 0.4387 with a standard error of 0.3437. The Wald and the likelihood ratio statistics associated with this variable are 1.6294 (p = 0.2018) and 1.6254 (p = 0.2023) respectively. Both tests indicate that the effect of individual variable radiosensitizer is non-significant. The estimated accelerator factor is 1.5507 (exponentiated value of parameter 0.4387). This means that after controlling for the age of the patient, the probability of a patient treated with "therapy with radiosensitizer misonidazole" surviving *t *days equals to the probability of a patient treated with "therapy without radiosensitizer misonidazole" surviving 1.5507 *t *days. For instance, the hypertabastic accelerated failure time model suggests that for 49 year old patients (median age for all patients under study), the probability of a patient treated with " therapy with radiosensitizer misonidazole " surviving 293 days equals to the probability of a patient treated with" therapy without radiosensitizer misonidazole " surviving 454 days. The Wald statistic for the age covariate is 22.18 (p < 0.0001).

**Table 3:** Statistical results for the hypertabastic accelerated failure time model and four comparison models for glioma brain cancer data

	-2Log likelihood	AIC	Estimate	SE	Chi-square	p-value
Hypertabastic	68.72	76.72				
*α*			0.0001312	0.0001956	0.45	0.4978
*β*			0.8343	0.1434	33.71	<0.0001
Radiosensitiser			0.4387	0.3437	1.63	0.2018
Age			0.0963	0.0205	22.18	<0.0001
Weibull	67.54	75.54				
*α*			0.0000003	0.0000007	0.12	0.2741
*β*			1.4203	0.2456	33.44	<0.0001
Radiosensitiser			0.3614	0.3040	1.41	0.2345
Age			0.0977	0.0197	24.61	<0.0001
Log-normal	71.15	79.15				
*α*			0.0000173	0.0000188	0.85	0.3563
*β*			0.9853	0.1501	43.10	<0.0001
Radiosensitiser			0.4498	0.3889	1.37	0.2427
Age			0.1003	0.0207	23.58	<0.0001
Log-logistic	70.45	78.45				
*α*			-19.6913	3.6445	29.19	<0.0001
*β*			1.8338	0.3251	31.82	<0.0001
Radiosensitiser			0.5000	0.3718	1.81	0.1787
Age			0.0942	0.0208	20.52	<0.0001
Cox	99.43	107.43				
Radiosensitiser			0.553	0.448	1.53	0.2169
Age			0.150	0.036	16.87	<0.0001

**Figure 6:**
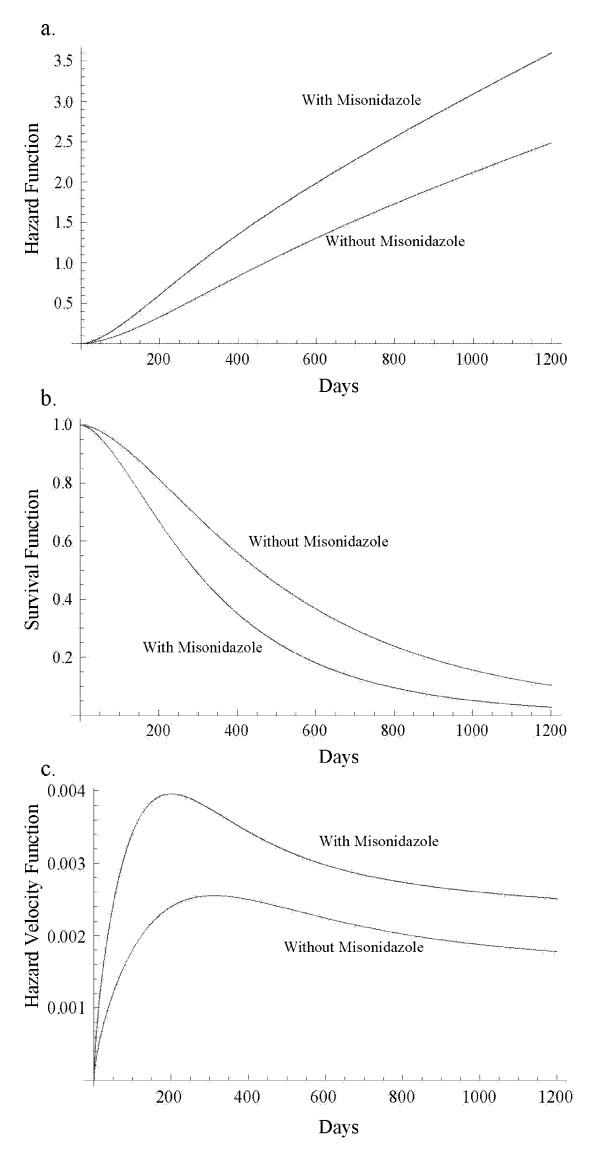
a) Hypertabastic hazard curves for glioma brain cancer; b) Hypertabastic survival curves for glioma brain cancer; c) Hypertabastic velocity of hazard curves for glioma brain cancer.

**Figure 7:**
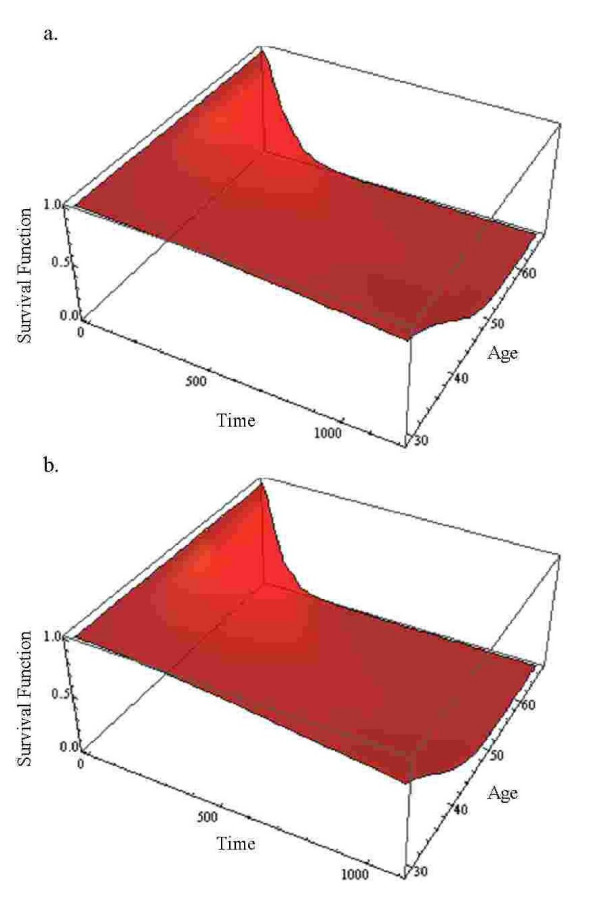
a) Hypertabastic 3D survival curve with variables time and age for group using radiotherapy with radiosentisizer misonidazole for glioma brain cancer; b) Hypertabastic 3D survival curve with variables time and age for group using radiotherapy without radiosentisizer misonidazole for glioma brain cancer.

Using AIC, we came to the conclusion that both Weibull and hypertabastic models fit the data very well. However, the Weibull AIC value was 75.54 which indicates the best fit. For the hypertabastic model, the AIC value was 76.72 which was slightly less than Weibull. The remaining AIC values for log-normal, log-logistic and Cox are 79.15, 78.45, and 107.43 respectively. At the median age of 49, the median survival time for those patients who underwent radiotherapy with radiosensitizer misonidazole using the hypertabastic, Weibul, log-normal, log-logistic, and the Cox models are 289, 316, 270, 277, and 244 days respectively. The corresponding median survival times for the group without the radiosensitizer misonidazole are 449, 453, 423, 456, and 488 days respectively. By examining the Kaplan-Meier survival functions for the two groups we observe the crossing pattern which is a clear indication of violation of proportional hazards assumption. Figures 6a and 6b show the graphs of hypertabastic hazard and survival functions for both treatment groups at the age level of 49. The hazards for both groups are increasing function of time. For those patients who received radiotherapy treatment with radiosensitizer misonidazole, hazard function reached its maximum velocity in about 200 days. For those who did receive radiotherapy without misonidazole, hazard function reached its maximum velocity in about 311 days. These are the points in time where the speeds of failure rates (hazards) are highest (Figure 6c). Figures 7a and 7b represent 3-dimensional graphs of survival by time and age for each treatment group separately.

## 9. Discussion and conclusion

In this paper we have introduced a new survival model called hypertabastic survival model. The overall results of our simulation indicate that the hypertabastic model performs best when the data is generated from the Weibull distribution. When the data comes from the gamma distribution, both the Weibull and hypertabastic distributions perform well. In the case when we generate survival data from the log-normal distribution, the log-logistic and hypertabastic distributions perform well but the gamma and Weibull perform poorly. In the last case, the Weibull distribution performance is the worst. We believe that the hypertabastic survival model is to some extent robust with respect to variations in the distribution. We believe that the hypertabastic hazard function can play a role in modeling failure rates in medical, biological and engineering fields. The hypertabastic model can also assist physicians and clinicians with their treatment planning through its ability to predict patients' outcome as well as the risk of disease recurrence. Gilbert et al. [30] argued that the pattern of instantaneous risk over time is more interesting than the cumulative risk. For instance, in a cancer study where recurrence time of malignant tumor is of interest, a bimodal hazard curve may represent the elevated incidences of early and late recurrences and the magnitude of the hazard rates at the peaks may reveal the intensity of the failure rates. Therefore we recommend that clinicians, practitioners and analysts consider using this model along with other models, and compare it to the models they ordinarily use before making any decision as to which model would provide the best fit and prediction.

## Competing interests

The author(s) declare that they have no competing interests.

## Authors' contributions

The survival model equations were developed by MAT and ZB based on their previous work on hyperbolastic growth and survival models. Applications of the models to data were done by MAT, ZB, DKW and KPS. Writing of the manuscript was done by MAT and ZB, and final edits by DKW and KPS.

## Supplementary Material

Additional file 1 Hypertabastic model fitting to multiple myeloma data using SAS PROC NLP. SAS PROC NLP code provided here demonstrates how to fit hypertabastic model to multiple myeloma data using both *time *and *log(time)*. Click here for file
