# Supplementary material for: Hypertabastic survival model
Source: Theor Biol Med Model. 2007 Oct 26;4:40. doi: 10.1186/1742-4682-4-40 (PMC2169222; doi:10.1186/1742-4682-4-40)
Supplement: Additional file 1 — Hypertabastic model fitting to multiple myeloma data using SAS PROC NLP. SAS PROC NLP code provided here demonstrates how to fit hypertabastic model to multiple myeloma data using both time and log(time). [file 1742-4682-4-40-S1.doc]

**Additional files**

Additional file 1

File format: DOC

Title: Hypertabastic model fitting to multiple myeloma data using SAS PROC NLP.

Description: SAS PROC NLP code provided here demonstrates how to fit Hypertabastic model to multiple myeloma data using both *time* and *log(time)*.

title 'Multiple Myeloma data';

title2 'Hypertabastic Model - time';

**proc** **nlp** data=myeloma tech=quanew cov=**2** vardef=n pcov phes maxiter=**250**;

max logf;

parms a=**.01**, b=**.1**, c=**1**, d=-**0.01**;

in1=time**b;

in2=tanh(in1);

in3=a-a*(in1)/in2;

in4=in3/b;

in5=tanh(in4);

in6=exp(c*logbun+d*hgb); /* covariates */

/* likelihood equation */

s= log(**1**/cosh(a*(**1**-in1/tanh(in1))/b)) * in6 +

y*Log (( a*(time**(-**1**+b)) * ( -**1**/tanh (in1) +

in1/sinh(in1)****2** ) * in5 ) * in6);

logf=s;

**run**;

title 'Multiple Myeloma data';

title2 'Hypertabastic Model - log time';

**proc** **nlp** data=myeloma tech=quanew cov=**2** vardef=n pcov phes maxiter=**250**;

max logf;

parms a=**.01**, b=**.1**, c=**.01**, d=-**0.1**;

in1=time**b;

in2=tanh(in1);

in3=a-a*(in1)/in2;

in4=in3/b;

in5=tanh(in4);

in6=exp(c*logbun+d*hgb); /* covariates */

/* likelihood equation */

s= log(**1**/cosh(a*(**1**-in1 * **1**/tanh(in1))/b)) * in6 +

y*Log (( a*(-**1***in1 * **1**/tanh (in1) + time**(**2***b) *

**1**/sinh(in1)****2** ) * in5 ) * in6);

logf=s;

**run**;
